# Changes in self-reported physical and mental health, behaviour and economic status among adults by known seropositivity and sociodemographic factors before and after the COVID-19 pandemic outbreak in Ischgl, Austria

**DOI:** 10.3389/fpubh.2025.1488108

**Published:** 2025-04-02

**Authors:** Katie Bates, Wegene Borena, Martin McKee, Lore Hayek, Paul Bouanchaud, Zoltán Bánki, Lydia Riepler, Annika Rössler, Barbara Falkensammer, Jörg Paetzold, Andreas Walser, Sebastian Schönherr, Lukas Forer, Ludwig Knabl, Janine Kimpel, Dorothee von Laer, Hanno Ulmer

**Affiliations:** ^1^Institute of Clinical Epidemiology, Public Health, Health Economics, Medical Statistics and Informatics, Medical University of Innsbruck, Innsbruck, Austria; ^2^Institute of Global Health, University College London, London, United Kingdom; ^3^Institute of Virology, Medical University of Innsbruck, Innsbruck, Austria; ^4^Faculty of Public Health and Policy, London School of Hygiene and Tropical Medicine, London, United Kingdom; ^5^Department of Political Science, University of Innsbruck, Innsbruck, Austria; ^6^Population Services International, London, United Kingdom; ^7^Center for Virology and Vaccine Research, Beth Israel Deaconess Medical Center, Harvard Medical School, Boston, MA, United States; ^8^Department of Economics, University of Salzburg, Salzburg, Austria; ^9^Walser's Surgery, Ischgl, Austria; ^10^Institute of Genetic Epidemiology, Medical University of Innsbruck, Innsbruck, Austria; ^11^Tyrolpath Obrist Brunhuber GmbH, Hauptplatz, Zams, Austria

**Keywords:** self-reported health, SARS-CoV-2 infection, pandemic (COVID-19), mental health, physical health, economic impacts, behaviour

## Abstract

**Introduction:**

In early March 2020, a SARS-CoV-2 outbreak occurred in the ski resort of Ischgl, in Austria. After an initial seroprevalence study in April 2020, a follow-up study in November 2020 showed persistence of high levels of seropositivity. The impacts of SARS-CoV-2 infections and non-pharmaceutical interventions required to reduce transmission were wide-ranging, including worsened mental and physical health and economic damage.

**Methods:**

We analysed data from the Ischgl follow-up study. Of the 1,259 adults that participated in the Ischgl-1 study (Ischgl-1), 801 were followed-up. Seropositivity was defined using presence of binding and neutralizing antibodies at Ischgl-1. At follow-up, 7 months later (Ischgl-2), participants reported changes to self-rated mental and physical health, physical activity, alcohol consumption, smoking and economic status. Changes were compared by serological status, using multivariable logistic and multinomial regression models, where appropriate, and adjusting for factors including age, sex, and morbidity.

**Results:**

1 in 2 participants reported experiencing a moderate or severe impact of the pandemic. One fifth of participants reported a worsening in their mental health from November 2019 to November 2020; women and participants aged ≥35 to <70 years were disproportionately affected. Seropositivity was associated with a decline in physical health but no decline in mental health or behaviour changes. Very few participants reported any changes in behaviours. The overriding impact the population of Ischgl was economic—50% of participants reported a worsening of their professional and/or financial situation. Declines in self-reported mental health were associated with the overall experience of the pandemic and economic factors.

**Conclusion:**

The population of Ischgl demonstrated a high level of resilience to the pandemic as measured by health. However, certain segments of the population were disproportionately affected, particularly with regard to mental health and economic wellbeing. Future pandemic preparedness must consider how pandemic mitigation strategies can be responsive to context and the wider impacts on mental health and social and economic wellbeing while minimising mortality and safeguarding health systems.

## 1 Introduction

The COVID-19 pandemic had direct and indirect effects on health. Those infected faced increased risk of severe illness (including Long COVID) and death and the resulting pressure on health facilities reduced access for those with other conditions. The non-pharmacological interventions (NPIs) necessary to interrupt transmission of the virus (1), including unprecedented restrictions on daily life, had wider consequences for the health, social and economic situation of individuals and populations (2, 3). These NPIs included travel restrictions, stay-at-home orders, school closures, social distancing, quarantine, and personal protective measures such as face masks and health system strengthening (4).

There are now many studies documenting these direct and indirect health impacts and their uneven distribution within populations. The German National Cohort (NAKO) study measured changes in self-rated health (SRH) among *n* = 113,928 participants from baseline (between 1 and 5 years prior to 2020) until late April to May 2020, collected in the COVID-NAKO survey. SRH improved for 32% of people but worsened for 12%. Those who were tested for the SARS-CoV-2 virus were more likely to report worsening self-rated health than those who did not, regardless of whether the result was positive or negative, suggesting that the impact of the pandemic on self-rated health may have been indirect (5). In an online survey in Austria, undertaken very early in the pandemic (March to April 2020), 43.3% of respondents rated the psychological impact of the pandemic as moderate or severe (6); women, older adults, and those with poor self-rated health reported higher psychological burdens related to the pandemic.

The term COVID-19 syndemic has been coined to describe the association of direct and indirect health impacts with pre-existing health and socioeconomic inequalities (7). For example, unemployed people in Sweden experienced worse economic (2), while in a multicounty study including the UK, Italy and Slovenia, economic impacts were more pronounced among younger (18–24) and older adults (65+), as well as those with lower levels of education and casual employment status (8). Data from the few countries to measure ethnicity also found marked inequalities (9).

The existing research on mental health impacts of the COVID-19 pandemic are complex and context-dependent, influenced by factors such as levels of support and local incidence rates of SARS-CoV-2. The NAKO Study revealed worse mental health outcomes among those tested for the virus, regardless of test results, with less pronounced effects in low-incidence areas and greater impacts in higher-incidence regions (5). A consistent finding in studies, including the SHARE COVID-19 study, is the presence of increased stress across all age groups, with women experiencing greater mental health declines than men (10). However, anxiety and depression were particularly prevalent among people under 60, especially those aged 20–39, aligning with similar trends observed in the UK and the US (5, 11, 12).

Initially, most studies examining the pandemic’s impact on individuals focussed on direct health effects and relied on data from groups for which there were good data, such as health care workers (13), or patients hospitalised with COVID-19 (14). Later studies, in particular those looking at indirect effects, have used data from app or web-based surveys, all subject to potential bias from participants’ self-selection and/or lacking laboratory confirmation of SARS-CoV-2 infection (15). Some longitudinal household surveys have been exploited to assess the effects of SARS-CoV-2 by adding in serological testing or asking for self-reported SARS-CoV-2 status (16). Whilst useful, more recent studies are limited as participants may have been infected in different waves of the pandemic, with different variants of SARS-CoV-2, in different areas of a country that may have experienced different NPIs, different levels of support, and different epidemiological contexts.

This paper reports findings from the Ischgl study in Austria. Ischgl, a popular area for ski tourism, experienced an outbreak of SARS-CoV-2 in early March 2020. The virus then spread further, mainly to Northern Europe and the US (17–19). An initial population survey, including epidemiological and seroprevalence data, was conducted in April 2020 (Ischgl-1). A follow-up study was conducted in November 2020 (Ischgl-2), following the start of the second wave in Austria in October 2020.

The first signs of the outbreak emerged around 5^th^ March 2020, when the international community pointed to Ischgl as an area of concern. Bars in the area closed on 10^th^ March after cases were linked to an apres-ski bar. On 13^th^ March the Paznaun valley, in which Ischgl is located, was placed into a sudden lockdown by the Federal Chancellor. The lockdown was announced in a press conference at 2 pm and police controls began 2 h later. Residents of Ischgl and foreign guest workers were obliged to stay in the area, but tourists were allowed to leave. This initial lockdown lasted until 23^rd^ April 2020 (20, 21).

After April 2020, Ischgl was subject to the same package of NPIs as the rest of Austria. Between March and November 2020, this included (at differing levels of severity over time) stay-at-home orders, working from home (where possible), school closures, restrictions on gatherings, cancellation of public events, restrictions on internal movement, testing and contract tracing, quarantine and isolation of SARS-CoV-2 cases and suspected cases, international travel restrictions and mask mandates, supported by public information campaigns, income support for people and businesses and health system strengthening (ICU management, purchase of personal protective equipment) (4, 22).

Despite the proliferation of research into the effects of the COVID-19 pandemic on a range of outcomes, the Ischgl follow-up study offers unique insights. The study was undertaken in an immunologically naïve population and provided laboratory-confirmed evidence of infection with follow-up in a population in which over 40% of the adult population was infected. Compared to similar municipalities fewer new infections were reported in Ischgl between the two waves of the survey (23). Capitalising on this unique situation, this study can compare a wide range of outcomes of the COVID-19 pandemic in a broadly static population exposed to the same NPIs in people with and without laboratory-confirmed evidence of infection.

This study aims to assess the impact of the COVID-19 pandemic on self-reported physical and mental health, behaviours, and economic status in Ischgl, Austria. As such, the study aims to contribute to a wider understanding of disparate effects of the pandemic across demographic and socioeconomics groups as well as understanding levels of resilience within a community heavily reliant on tourism. In doing so, the study aims to provides insights that are relevant for developing pandemic preparedness and recovery strategies.

## 2 Methods

### 2.1 Overview

Using single-item measures of self-rated physical health (SRPH) and self-rated mental health (SRMH) widely used in research on inequalities in health in European contexts, this study reports changes in these measures by seropositivity before and after the pandemic (24, 25) The study also assesses self-reported changes in behaviours and economic circumstances before and after the pandemic, by known seropositivity after the SARS-CoV-2 outbreak, and by sociodemographic and socioeconomic factors. In this way, it aimed to assess the association of seropositivity with the outcomes studied and any disparities in the impacts of the pandemic.

### 2.2 Study population, study design and recruitment

After the initial outbreak of SARS-CoV-2, we conducted an initial survey (“Ischgl-1”), with a follow-up study in November 2020 (“Ischgl-2”) (23, 26). Ischgl-1 was a cross-sectional epidemiological survey with measurement of seroprevalence targeted all residents of Ischgl/Tyrol regardless of age or gender. It was conducted between April 21st and 27th, 2020. The study was approved by the ethics committee (EC) of the Medical University of Innsbruck with EC number 1100/2020 (Ischgl-1). 1,259 adults, participated in Ischgl-1, corresponding to around 80% of those living in the town at that time (both permanent residents and seasonal workers). By the end of April 42% of the local population (45% of the adult population) were seropositive, one of the highest seroprevalence levels reported in spring 2020 worldwide (26). The follow-up study, Ischgl-2 (EC Number 1330/2020), was conducted between November 2nd and 8th, 2020.

The current sample comprises the 801 adults aged 18 and over who participated in both Ischgl-1 and Ischgl-2. In Ischgl-1, 1,527 adults were invited to participate, of whom 1,259 (82.4%) agreed (26). 813 (64.6%) of them participated in the follow-up study, Ischgl-2, but 12 were excluded from the analysis due to inconsistent recording of age (*n* = 9), failure to complete a questionnaire (*n* = 2) and no blood sample (*n* = 1). The remaining 801 included slightly more (50.2% vs. 45%) who had been seropositive and more women (54% vs. 51.9%). The age distribution was similar in both studies (23).

### 2.3 Materials and methods

The materials and methods used to test for SARS-CoV-2 antibodies and generate an indicator of previous infection with SARS-CoV-2 have been described elsewhere (23). In brief, blood samples were analysed for SARS-CoV-2-binding antibodies using four immunoassays. Samples were screened for anti-SARS-CoV-2-S1-protein IgA and IgG positivity by a commercially available anti-SARS-CoV-2-IgA and-IgG ELISA (Euroimmun, Lübeck, Germany), respectively, using the fully automated 4-plate benchtop instrument Immunomat™ (Virion/Serion, Würzburg, Germany). For both assays values >1.1 were considered positive. Borderline values (0.8–1.1) in the Euroimmun IgG ELISA were rated positive; for the Euroimmun IgA ELISA borderline values were rated as negative. Samples were additionally immunoassayed for anti-SARS-CoV-2 N-protein IgG (anti-N IgG) antibodies in a fully automated manner on the ARCHITECT i2000SR system (Abbott, Illinois, USA). Samples were considered positive if the detected relative light unit (RLU) was >1.4. Anti-N IgG was also quantified using the ElecsysAnti-SARS-CoV-2 (Roche Diagnostics, Indianapolis, USA) according to manufacturer’s recommendations. A COI of ≥1.0 was considered positive. Titres of SARS-CoV-2 neutralizing antibodies were determined using a replication defective vesicular stomatitis virus (VSV) pseudotyped with SARS-CoV-2 spike protein Titers of ≤1:4 were considered as negative, titres of ≥1:16 as positive.

### 2.4 Defining seropositivity

Plasma samples from Ischgl-1 were analysed. The serostatus of the samples was defined as p, d, a or n depending on the binding antibody assays: p = positive = anti-S IgG+ AND anti-N IgG+ (either Roche or Abbott assay positive); d = discordant = anti-S IgG+ OR anti-N IgG+ (either Roche or Abbott assay); a = only IgA+ = only anti-S IgA+ but anti-S IgG-AND anti-N IgG-; *n* = negative = anti-S IgG-AND anti-N IgG-AND anti-S IgA-. To calculate the seroprevalence, all individuals with serostatus p were considered as seropositive. Individuals with ‘d’ and ‘a’ samples were considered as seropositive if they had neutralizing antibodies ≥1:16.

### 2.5 Questionnaires

Ischgl-2 participants were interviewed to answer a questionnaire which included questions about changes to self-rated physical and mental health between November 2019 and November 2020 (Ischgl-2), pre-existing medical conditions, selected medications and symptoms (from a pre-specified list but with the ability to specify other symptoms) including their duration since April 2020 (Ischgl-1) (Table 1). The questionnaire was designed to generate data comparable with existing surveys, including the Community Response Survey developed by John Hopkins University (JHU CRS) that was specifically developed for assessing the impacts of COVID-19 (27), as well as other research on COVID-19 symptoms known about at the time, modified for the Austrian context and translated into German.

**Table 1:** Overview of questionnaire Data collected at Ischgl-1 and Ischgl-2.

Ischgl-1 Questionnaire (April 2020):
All Participants: Sociodemographic characteristics Household Characteristics Symptom onset (date) Symptoms
Ischgl-2 Questionnaire (November 2020):
All participants: Current sociodemographic characteristics Self-reported SARS-CoV-2 status (previously infected or not) Current symptoms Symptoms experienced since Ischgl-1 PCR testing/SARS-CoV-2 antibody tests since Ischgl-1 Current socioeconomic status (education, employment, economic status) Self-rated change in socioeconomic status between Summer 2019 (2019 low season) and Ischgl-2 (November 2020) Current self-reported morbidity at Ischgl-2 Current behaviours (smoking, alcohol, diet, physical activity) at Ischgl-2 Changes in behaviours (smoking, alcohol, diet, physical activity) between first lockdown (mid-March 2020) and Ischgl-2 Self-rated physical and mental health at Ischgl-2 Changes in self-rated physical and mental health in last year (November 2019 to November 2020) Overall impact of pandemic Participants reporting positive PCR/antibody test for COVID-19*: Symptoms of SARS-CoV-2 infection Duration of COVID-19 symptoms Episodic nature of COVID-19 symptoms Other symptoms Most problematic symptom Longest symptom

### 2.6 Data collection

Data were collected using Askimed, a web-based eCRF system for data collection and management. The system included checks for completeness, internal consistency, and validity. All interviewers attended a pre-fieldwork training session on the questionnaire and the Askimed system [Askimed access date: 20. 07. 2021 (Available online at: https://www.askimed.com/)].

### 2.7 Outcomes measures/dependent variables

Seropositivity was defined according to status at Ischgl-1 while all outcome measures were defined using data collected at Ischgl-2. These outcome measures were:

#### 2.7.1 Self-reported decline in physical and mental health

At Ischgl-2, all participants were asked to report if their physical or mental health had declined or improved between November 2019 and November 2020 (the last year) on a 5-point scale. The data were dichotomised into declined = 1 (“a lot worse,”” a little worse”) and otherwise = 0 (“no change, “a little better,” “much better”). All participants were also asked to self-report their current physical (SRPH) and mental health (SRMH) at Ischgl-2 by rating them separately on separate single-item 5-point scales (from poor to excellent). For analyses, SRPH and SRMH were dichotomised to poor = 1 (“poor,” “fair”) and good = 0 (“good,” “very good,” “excellent”).

#### 2.7.2 Changes in smoking, alcohol consumption and physical activity

Participants were asked to report if they currently smoke, consume alcohol and exercise and if these behaviours had increased or decreased consumption since the onset of the first lockdown in Ischgl (13^th^ March 2020). For exercise, participants were asked about changes in both frequency and intensity. Categorical variables were constructed for changes in each behaviour (“stayed the same,” “increased,” “decreased”). Additionally, people were asked if their weight had stayed the same, increased or decreased since lockdown. Those who had experienced a change in weight were asked to self-report, in kilograms, their change.

#### 2.7.3 Symptoms since Ischgl-1

Participants were asked to report any new symptoms they experienced since Ischgl-1 (April 2020), whether these were episodic in nature, their duration (in days) and whether they were still experiencing these symptoms.

#### 2.7.4 Economic impact of the pandemic

Participants rated changes in their individual financial and professional situation since summer 2019 on a 5-point scale; data for both variables were dichotomised to worse = 1 (“significantly worse,” “slightly worse”) and same/improved = 0 (“no change,” “slightly improved,” “much improved”) and the extent to which they agreed that they were concerned about their future financial and professional security agree = 1 (“completely agree,” “agree”) and otherwise = 0 (“neither agree nor disagree,” “disagree,” “strongly disagree”). Summer 2019 was chosen to reflect the natural peaks and troughs in the tourism industry, which is a main source of economic activity in Ischgl.

#### 2.7.5 Overall Impact of the pandemic

Participants were asked to rate, on a 4-point scale, the overall impact of the pandemic on their daily lives, a question taken from the JHU CRS (27); data were dichotomised (“moderately/severely affected” = 1 and “a little affected, completely unaffected” = 0).

### 2.8 Seropositivity at Ischgl-1

For the analyses presented here, seropositivity in Ischgl-1 is used to assess the relationship between prior SARS-CoV-2 infection and the outcome measures.

### 2.9 Statistical analysis

Summary characteristics are presented as means ± standard deviation (SD) or median and Interquartile Range (IQR), where appropriate, for continuous variables and number (n) and percentage (%) for categorical variables.

The relationships between the dependent variables and seropositivity were analysed using multivariate binary and multinomial logistic regression models, where appropriate. Age group (18 to <35 years, ≥35 to <50 years, ≥50 to <70 years, and ≥ 70 years), sex and morbidity (binary variable of self-reported hypertension, diabetes, chronic kidney disease (CKD), cancer, cardiovascular disease (CVD), neurological diseases and other lung diseases = 1, or none) were controlled for in all models. Other covariates included body mass index [“normal or underweight” (<25 kg/m^2^), “overweight” (≥25 to <30 kg/m^2^) or obese (≥30 kg/m^2^)], education (Compulsory/High School/University/Other) and working in the tourism sector (yes/no) as well as the outcome measures outlined above used as covariates in models, where appropriate.

Adjusted odds ratios (ORs) from binary logistic regression models are reported and adjusted relative risk ratios (RRs) from multinomial logistic regression models are reported with 95% confidence intervals (95% CI). Statistical tests were two-tailed statistical significance was set at an alpha level of 0.05. All analysis was done using Stata version 17.1 (Stata Corp., College Station, TX, USA).

## 3 Results

### 3.1 Sample characteristics

Table 2 presents the characteristics of participants in the follow-up sample (*n* = 801). Just over 50% were seropositive in Ischgl-1 (50.3%, *n* = 403), 54.9% were female (*n* = 440), median age was 45 years (SD 15.8), one quarter reported at least one specified morbidity (25.7%, *n* = 205) and over 40% were overweight (BMI ≥ 25 kg/m^2^, *n* = 331). There was no significant difference in seropositivity by sex, age, morbidity or BMI in the sample.

**Table 2:** Characteristics of follow-up sample (Ischgl-2) by seroprevalence at Ischgl-1.

	Seropositive Ischgl-1	Seronegative Ischgl-1	Total
Total, *n* (%)			
	403 (50.3)	398 (49.7)	801 (100)
Sex, *n* (%)			
Female	210 (52.1)	230 (57.8)	440 (54.9)
Male	193 (47.9)	168 (42.2)	361 (45.1)
Age group (years), *n* (%)			
18 to <35	114 (28.3)	114 (28.6)	228 (28.5)
≥35 to <50	122 (30.3)	107 (26.9)	229 (28.6)
≥50 to <70	140 (34.7)	146 (36.7)	286 (35.7)
≥70	27 (6.7)	31 (7.8)	58 (7.2)
Age (years) Median (IQR)	46 (32–57)	45 (33–58)	45 (33–57)
Morbidity^^, *n* (%)			
No	298 (74.1)	296 (75.6)	594 (74.3)
Yes	104 (25.9)	101 (25.4)	205 (25.7)
BMI group (kg/m^2^), *n* (%)			
<25	226 (56.4)	239 (60.5)	465 (58.4)
≥25 to <30	129 (32.2)	123 (31.1)	252 (31.7)
≥30	46 (11.5)	33 (8.4)	79 (9.9)
Decline in Physical Health in last year^, *n* (%)			
Yes	47 (11.7)	22 (5.5)	69 (8.6)
No	356 (88.3)	375 (94.5)	731 (91.4)
Current Self-reported Physical Health^, *n* (%)			
Poor	24 (6.0)	13 (3.3)	37 (4.6)
Other	379 (94.0)	384 (96.7)	763 (95.4)
Decline in Mental Health in last year^, *n* (%)			
Yes	96 (23.8)	90 (22.7)	186 (23.3)
No	307 (76.2)	307 (77.3)	614 (76.8)
Current Self-reported Mental Health^, *n* (%)			
Poor	53 (13.2)	54 (13.6)	107 (13.4)
Other	350 (86.9)	343 (86.4)	693 (86.6)

^*n* = 800, data missing for one study participant; ^^morbidity defined as any of: self-reported hypertension, diabetes, chronic kidney disease (CKD), cancer, cardiovascular disease (CVD), neurological diseases and other lung diseases.

**Figure 1:**
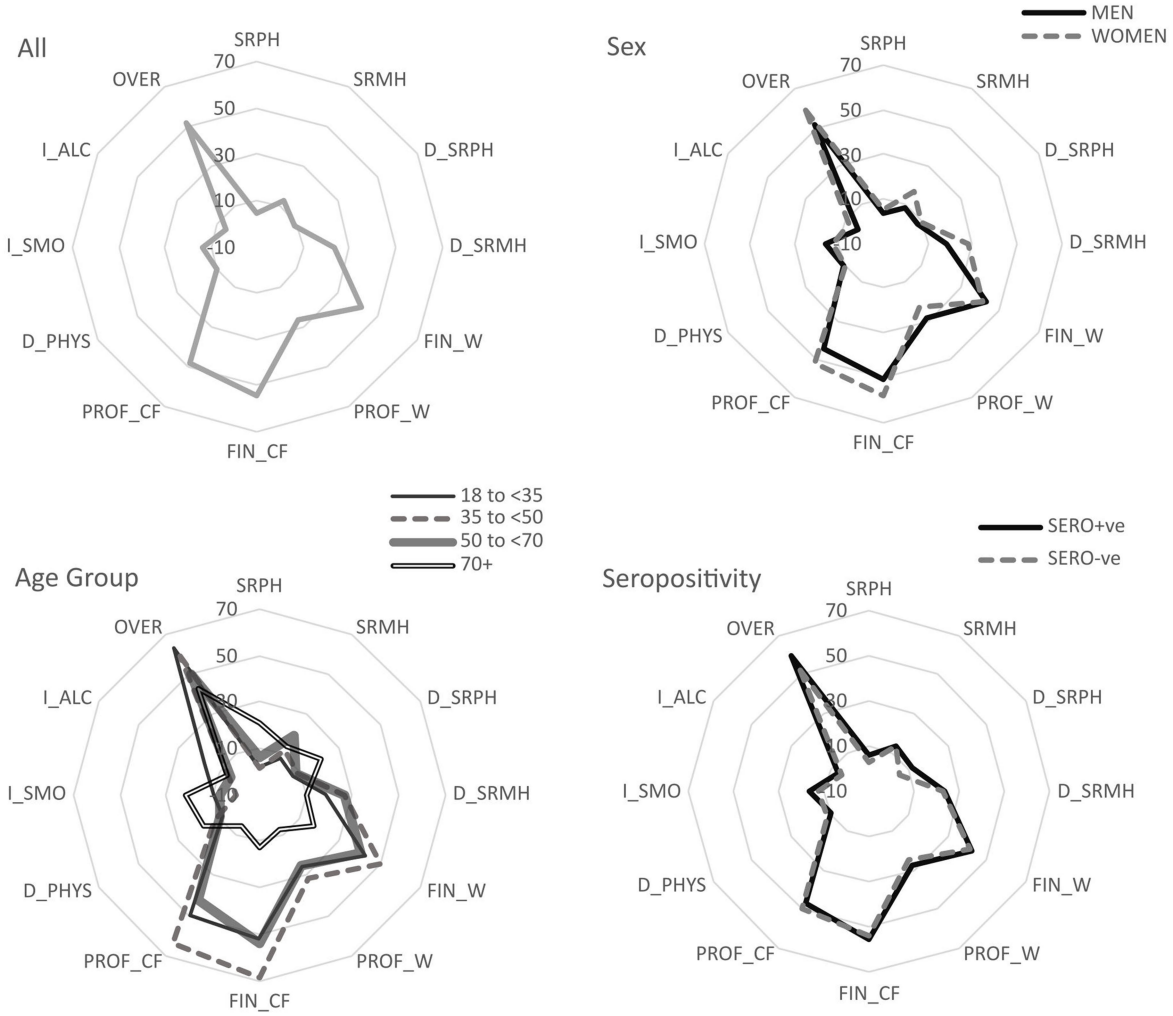
Overview of percentage of participants reporting negative outcomes of the pandemic for each outcome measure, overall and by subgroups. Key: SRPH: self-rated physical health, SRMH: self-rated mental health, D_SRPH: decline in self-rated physical health compared to last year, D_SRMH: decline in self-rated mental health compared to last year, I-ALC: increase in alcohol consumption, I_SMO: increase in smoking, D_PHYS: decline in physical activity, OVER: overall moderate/severe effect of the pandemic, FIN_W: financial situation worse since last summer, PROF_W: professional situation worse since last summer, FIN_CF: completely/strongly agree concerned about future financial situation, PROF_CW: completely/strongly agree concerned about future professional situation. Smoking, Alcohol and Physical Activity % are among people who have ever smoked/consumed alcohol or exercised, not whole sample.

Figure 1 shows the total percentage of participants reporting negative impacts across all outcome measures in November 2020 by sex, age and seropositivity. Over 55% of participants reported the pandemic had a moderate or severe impact on them. An age gradient was apparent; 63.2% of 18 to <35-year-olds reported a moderate/severe effect of the pandemic compared to 42.9% of adults ≥70 years. A greater proportion of participants reported worse economic outcomes than health outcomes (including negative impacts on behaviours). 8.6% of all participants reported a decline in physical health and nearly a quarter reported a decline in their mental health between November 2019 and November 2020 (the last year). Nearly half of all participants were concerned about their future professional situation (48.4%); the highest proportion of concern was reported among adults aged ≥35 to <50 years (63.9) and the lowest among those aged ≥70 years (5.5%). Similarly, 45.4% of participants were concerned about their future financial situation, with 68.7% of those aged ≥35 to <50 years concerned but only 12.5% of those aged 70 years and over. Only 5.3% of all participants increased alcohol consumption, and only 8.6% of all participants reported a decline in their physical health since the first lockdown implemented in Ischgl in mid-March 2020.

### 3.2 Results for seropositivity and changes in outcome measures

#### 3.2.1 Decline in self-rated physical health

Those in the seropositive group were more than twice as likely to report a decline in physical health than the seronegative (11.7 and 5.5%, respectively, Table 3). After adjusting for age, sex, BMI, education and morbidity, seropositive participants remained more than twice as likely as the seronegative to report a decline in SRPH (OR 2.38 (95% CI: 1.39, 4.09), Table 4). Other associations seen in the bivariate analysis also remained similar after adjustment (Tables 3, 4).

**Table 3:** Decline in SRPH and decline in SRMH by seropositivity at Ischgl-1.

	Self-rated Decline in Physical Health in year preceding Ischgl-2^¥^		*p*-value	Self-rated Decline in Mental Health in year preceding Ischgl-2^¥^		*p-*value
	Yes	No		Yes	No	
Total *n* (%)	69 (8.6)	731 (91.4)		186 (23.3)	614 (76.8)	
Seropositivity at Ischgl-1, *n* (%)						
Seropositive	47 (11.7)	356 (88.3)	0.002	96 (23.8)	307 (76.2)	0.700
Seronegative	22 (5.5)	375 (94.5)		90 (22.7)	307 (77.3)	
Sex, *n* (%)						
Female	40 (9.1)	399 (90.9)	0.589	121 (27.6)	318 (72.4)	0.001
Male	29 (8.0)	332 (92.0)		65 (18.0)	296 (82.0)	
Age Groups years, *n* (%)						
18 to <35	15 (6.6)	213 (93.4)	0.007	42 (18.4)	186 (81.6)	0.007
≥35 to <50	20 (8.7)	209 (91.3)		61 (26.6)	168 (73.4)	
≥50 to <70	22 (7.7)	263 (92.3)		77 (27.0)	208 (73.0)	
≥70	12 (20.7)	46 (79.3)		6 (10.3)	52 (89.7)	
Age group (≥/<median), *n* (%)						
≥45	42 (10.1)	373 (89.9)	0.118	101 (24.3)	300 (77.9)	0.450
<45	27 (7.0)	358 (93.0)		85 (22.1)	314 (75.7)	
BMI group (kg/m^2^)^†^, *n* (%)						
<25.0	36 (7.7)	429 (92.3)	0.473	118 (25.4)	347 (74.6)	0.238
≥25.0 to <30.0	24 (9.6)	227 (90.4)		50 (19.9)	201 (80.1)	
≥30.0	9 (11.4)	70 (88.6)		17 (21.5)	62 (78.5)	
Morbidity^††^, *n* (%)						
No (%)	30 (14.6)	175 (85.4)	<0.001	136 (22.9)	458 (77.1)	0.662
Yes (%)	39 (6.6)	555 (93.4)		50 (24.4)	155 (75.6)	
Current Self-reported Physical Health, *n* (%)						
Not Poor	45 (5.9)	718 (94.1)	<0.001	168 (22.0)	595 (78.0)	<0.001
Poor	24 (64.9)	13 (35.1)		18 (48.7)	19 (51.4)	
Current Self-reported Mental Health, *n* (%)						
No Poor	49 (7.1)	644 (92.9)	<0.001	91 (13.1)	602 (86.9)	<0.001
Poor	20 (18.7)	87 (81.3)		95 (88.8)	12 (11.2)	
Decline in Physical Health, *n* (%)						
No				155 (12.2)	576 (78.8)	<0.001
Yes				31 (44.9)	38 (55.1)	
Decline in Mental Health, *n* (%)						
No	38 (6.2)	576 (93.8)	<0.001			
Yes	31 (16.7)	155 (83.3)				

¥ Total *n* = 800.

† *n = 796 for SRPH and n = 795 for SRMH.*

†† Total *n* = 799. *p <0.05, **p <0.01, ***p <0.001.

**Table 4:** Adjusted odds ratios (OR) for reporting decline in SRPH and decline in SRMH at Ischgl-2.

	OR of reporting decline in SRPH at Ischgl-2 (95% CI)	OR of reporting decline in SRMH at Ischgl-2 (95% CI)
Seropositivity (ref: Seronegative) Seropositive	2.38** (1.39, 4.09)	1.08 (0.77, 1.51)
Sex (ref: Male)		
Female	1.33 (0.78, 2.27)	1.84** (1.29, 2.64)
Age (ref: ≥50 to <70 years)		
18 to <35	1.12 (0.53, 2.39)	0.53** (0.39, 0.86)
≥35 to <50	1.52 (0.76, 3.02)	0.95 (0.62, 1.45)
≥70	2.44* (1.08, 5.54)	0.29** (0.12, 0.73)
BMI (ref: 25.0 to 30.0 kg/m^2^)		
<25.0	0.82 (0.45, 1.48)	1.20 (0.81, 1.79)
>30.0	0.93 (0.40, 2.15)	0.95 (0.50, 1.79)
Morbidity (ref: None)		
Yes	2.20* (1.21, 4.00)	1.39 (0.91, 2.11)
Education (ref: Compulsory)		
Other	1.26 (0.33, 4.82)	12.02 (0.76, 5.33)
High School	0.82 (0.44, 1.55)	1.68* (1.07, 2.64)
Degree	1.44 (0.58, 3.54)	1.53 (0.78, 3.01)
*N*	794	794

*p <0.05, **p <0.01, ***p <0.001.

#### 3.2.2 Decline in self-rated mental health

23.3% reported that their mental health had declined in the last year (Table 3; Figure 1); 13.4% of participants rated their current mental health as poor (Table 5). In contrast to the situation with SRPH, there was no significant difference in the proportion reporting a decline in SRMH by seropositivity. This remained non-significant after full adjustment for the same variables as used with SRPH (Tables 3, 4).

**Table 5:** Adjusted odds ratios for economic outcome measures.

	OR of reporting professional situation worse since Summer 2019	OR of reporting concern about future professional situation	OR of reporting financial situation worse than Summer 2019	OR of reporting concern about future financial security
Seropositivity at Ischgl-1 (ref: Seronegative)				
Seropositive	0.99 (0.70, 1.38)	0.67* (049, 0.93)	0.92 (0.68, 1.25)	0.94 (0.69, 1.28)
Education (ref: Compulsory)				
Other	1.16 (0.40, 3.31)	0.56 (0.20, 1.55)	0.97 (0.37, 2.52)	0.46 (0.18, 1.20)
High School	1.05 (0.68, 1.63)	1.05 (0.70, 1.59)	1.30 (0.88, 1.94)	0.97 (0.65, 1.44)
Degree	1.81 (0.94, 3.45)	0.85 (0.46, 1.58)	2.07* (1.15, 3.73)	0.59 (0.33, 1.08)
Tourism sector worker (ref: No)				
Yes	7.28*** (3.83, 13.82)	4.88*** (3.16, 7.53)	2.64*** (1.75, 3.97)	2.69*** (1.83, 3.94)
Sex (ref: Male)				
Female	1.34 (0.95, 1.89)	1.27 (0.92, 1.75)	0.88 (0.65, 1.20)	1.25 (0.91, 1.71)
Age Groups years (ref ≥50 to <70 years)				
18 to <35	1.03 (0.66, 1.62)	1.52* (1.06, 2.44)	1.10 (0.73, 1.65)	1.13 (0.75, 1.69)
≥35 to <50	1.17 (0.72, 1.80)	2.25*** (1.49, 3.41)	1.26 (0.85, 1.86)	1.87** (1.24, 2.82)
≥70	0.54 (0.18, 1.68)	0.16** (0.05, 0.58)	0.60 (0.27, 1.31)	0.21** (0.09, 0.50)
Morbidity^††^(ref: No morbidity)				
Yes	0.95 (0.61, 1.47)	0.98 (0.65, 1.47)	0.87 (0.59, 1.28)	1.12 (0.76, 1.67)
Self-rated Physical Health (ref: Not poor)				
Poor	1.14 (0.42, 3.12)	0.50 (0.18, 1.37)	1.08 (0.45, 2.60)	0.85 (0.34, 2.12)
Self-rated Mental Health (ref: Not poor)				
Poor	0.77 (0.42, 1.39)	1.23 (0.66, 2.31)	0.92 (0.52, 1.63)	1.13 (0.60, 2.10)
Decline in Physical Health (ref: No decline)				
Yes	1.20 (0.62, 2.31)	1.14 (0.58, 2.21)	1.28 (0.69, 2.37)	1.06 (0.56, 2.03)
Decline in Mental Health (ref: No decline)				
Yes	1.68* (1.06, 2.67)	2.73*** (1.67, 4.44)	2.12** (1.35, 3.34)	2.61*** (1.59, 4.27)
*N*	797	792	767	794

*p <0.05, **p <0.01, ***p <0.001.

#### 3.2.3 Changes in behaviours

Data on current smoking, alcohol consumption and exercise are reported in Web Appendix 3. Overall, 32.3% of the participants currently smoke, 83.0% consume alcohol and 13.6% do not exercise. People who were seropositive at Ischgl-1 were less likely to report currently smoking than those who were seronegative [OR 0.62 (95% CI: 0.45, 0.84)]; no relationship between seropositivity and current alcohol consumption or exercise was found (Web Appendix 4). Seropositivity was not associated with changes in smoking, alcohol consumption or exercise patterns since the first lockdown in mid-March 2020.

#### 3.2.4 Weight changes

The majority of participants self-reported no change in weight (75.3%). In adjusted analyses, seropositivity was not associated with the risk of weight gain compared to stable weight but was associated with weight loss [RR 1.99, (95% CI: 1.11, 3.56)].

#### 3.2.5 Symptoms since Ischgl-1

**Figure 2:**
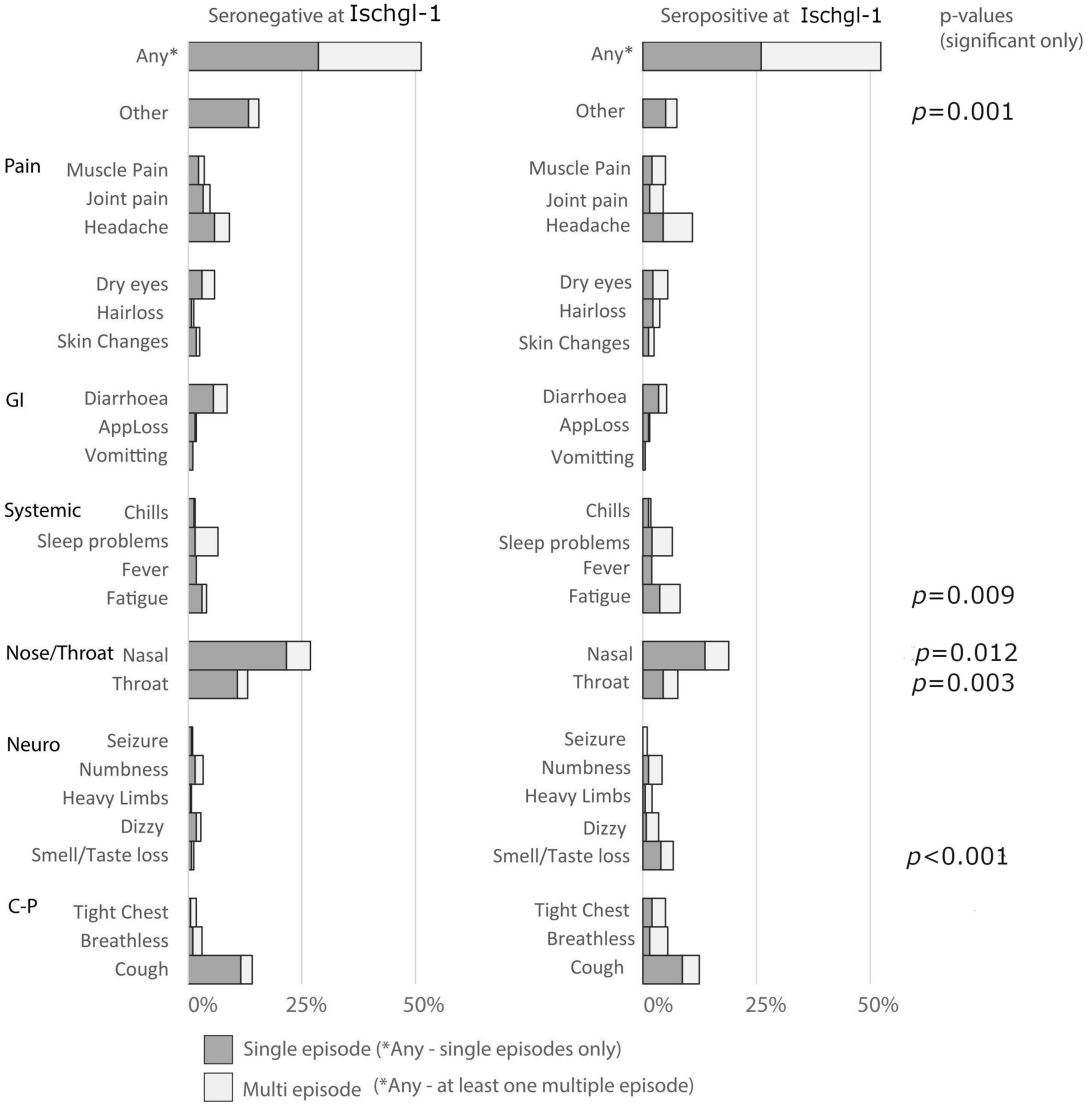
New onset symptoms since Ischgl-1. Key: GI: gastrointestinal, Neuro: Neurological, C-P: Cardiopulmonary.

Participants reported any new episodes of symptoms they experienced since Ischgl-1. For participants who were seropositive, this analysis excludes symptoms that participants attributed to their infection with SARS-CoV-2. Participants reported similar proportions of experiencing new symptoms (seropositive 52.4%, seronegative 51.3%, Figure 2) and similar numbers of different symptoms since Ischgl-1 (mean among seropositive—1.3 different symptoms (95% CI: 1.1, 5.2; mean seronegative 1.4 different symptoms [95% CI: (1.2, 1.5)].

People who were seropositive at Ischgl-1 were more likely to report new episodes of loss of taste and/or smell [OR 5.73 (95% CI: 2.16, 15.22)], tight chest [OR 2.91 (95% CI: 1.20, 7.06)] and fatigue [OR 2.07 (95% CI: 1.11, 3.86)] than seronegative participants. Seropositive participants reported fewer new episodes of nasal (runny/blocked nose) [OR 0.60 (95% CI: 0.42, 0.84)] and throat symptoms [OR 0.56 (95% CI: 0.35, 0.90)] than seronegative participants (Web Appendix 6). No other significant associations between seropositivity and specific symptoms were found among the sample. Hair loss was only reported among women (*n* = 20/440); among women being seropositive was associated with an increased odds of hair loss [OR 3.18 (95% CI: 1.12, 8.98)] compared to seronegative women.

#### 3.2.6 Change in economic outcomes and future concerns

In November 2020, over 1 in 4 adults in Ischgl considered their professional situation to be worse than the summer before (summer 2019, the last “normal” low season for tourism since the outbreak) and nearly half of all adults (48.4%) were concerned about their future professional situation. 42.1% of adults reported a worsening of their financial situation and 54.6% were concerned about how it might change in the future. Seropositivity was not significantly associated with changes in professional or financial situation, nor with concern about future finances. Participants seropositive at Ischgl-1 were a third less likely to be concerned about their professional future than seronegative participants (OR 0.67 (95% CI: 0.49, 0.93) Table 5).

#### 3.2.7 Overall impact of the pandemic

Overall, just over half of all participants reported that the COVID-19 pandemic had influenced their lives moderately or severely (55.6%, Web Appendix 8). In all models, being younger, experiencing a decline in mental health and worsened professional and financial situation were associated with reporting a moderate/severe impact of the pandemic overall (Models 1–4, Table 6). Seropositivity was only found to be significantly associated with the overall effect of the pandemic in multivariate analyses once controlling for concern for future professional and financial situation (Model 4, Table 6).

**Table 6:** Adjusted odds ratios for overall impact of pandemic vs. otherwise.

	OR for Self-reported Impact of Pandemic overall being Moderate/severe over not Model 1	OR for Self-reported Impact of Pandemic overall being Moderate/severe over not Model 2	OR for Self-reported Impact of Pandemic overall being Moderate/severe over not Model 3	OR for Self-reported Impact of Pandemic overall being Moderate/severe over not Model 4
Seropositivity at Ischgl-1 (ref: Seronegative)				
Seropositive	1.33 (0.98, 1.80)	1.33 (0.98, 1.81)	1.37 (0.99, 1.87)	1.38* (1.01, 1.90)
Education (ref: Compulsory)				
Other	1.53 (0.62, 3.80)	1.54 (0.62, 3.80)	1.48 (0.59, 3.69)	1.60 (0.64, 4.02)
High School	1.43 (0.97, 2.12)	1.43 (0.97, 2.12)	1.38 (0.92, 2.06)	1.40 (0.93, 2.09)
Higher Education	1.64 (0.90, 2.96)	1.64 (0.91, 2.97)	1.43 (0.78, 2.63)	1.53 (0.83, 2.83)
Works in tourism sector (ref: Works in other sector)				
Yes	1.47* (1.00, 2.15)	1.47* (1.00, 2.15)	1.14 (0.77, 1.70)	1.06 (0.71, 1.60)
Sex (ref: Male)				
Female	1.32 (0.97, 1.79)	1.31 (0.96, 1.79)	1.29 (0.94, 1.77)	1.27 (0.93, 1.75)
Age Groups (years) (ref: ≥50 to <70 years)				
18 to <35	1.91** (1.26, 2.89)	1.91** (1.26, 2.90)	1.94** (1.27, 2.96)	1.90** (1.24, 2.91)
≥35 to <50	1.44 (0.96, 2.15)	1.44 (0.96, 2.16)	1.41 (0.93, 2.12)	1.33 (0.88, 2.02)
≥70	0.92 (0.47, 1.80)	0.92 (0.47, 1.81)	0.98 (0.49, 1.92)	1.06 (0.54, 2.11)
Morbidity^††^ (ref: No morbidity)				
Yes	1.22 (0.83, 1.81)	1.22 (0.83, 1.81)	1.24 (0.83, 1.85)	1.22 (0.82, 1.83)
Decline in Self-rated physical health (ref: No decline)				
Yes	1.53 (0.84, 2.77)	1.54 (0.80, 2.96)	1.47 (0.75, 2.86)	1.49 (0.76, 2.92)
Decline in Mental Health (ref: No decline)				
Yes	3.85*** (2.55, 5.82)	3.69*** (2.20, 6.19)	3.36*** (1.98, 5.70)	3.15*** (1.85, 5.36)
Self-rated Physical Health				
Poor	-	0.97 (0.40, 2.38)	0.95 (0.39, 2.35)	0.96 (0.39, 2.39)
Self-rated Mental Health				
Poor	-	1.09 (0.57, 2.09)	1.14 (0.59, 2.20)	1.13 (0.58, 2.20)
Professional situation worsened				
Yes	-	-	2.05** (1.36, 3.10)	1.93** (1.27, 2.94)
Financial situation worsened				
Yes	-	-	1.54* (1.09, 2.16)	1.43* (1.00, 2.03)
Concern for Professional future				
Yes	-	-	-	1.09 (0.72, 1.64)
Concern for Financial Future				
Yes	-	-	-	1.40 (0.94, 2.07)
*N*	762	762	762	762

*p <0.05, **p <0.01, ***p <0.001.

### 3.3 Results for outcome measures by age, sex and working in tourism

All results reported are from adjusted analyses and control for seropositivity.

#### 3.3.1 Age

Those aged 18 to <35 years were significantly more likely to report a moderate or severe effect of the pandemic than those aged ≥50 to <70 years (OR 1.90 (1.24, 2.91) Model 4, Table 6). Whilst people 18 to <35 years had the lowest levels of current poor SRMH at Ischgl-2 (Web Appendix 1) nearly 1 in 4 reported a decline in MH since November 2019 (in the last year) (Table 3). This age group was 1.5 times as likely to be concerned for their future professional and economic circumstances than those aged ≥50 to <70 years (Table 5); the magnitude of the difference was even greater when the comparison was with those aged 70 years and above. Alcohol consumption since Ischgl-1 reduced in this age group compared to the others (Web Appendix 5). Those aged 18 to <35 years old were more likely to report nasal symptoms, throat symptoms and fatigue since Ischgl-1 than those aged ≥50 to <70 years (Web Appendix 6).

People aged ≥35 to <50 years were not more likely to report a moderate or severe impact of the pandemic than those aged ≥50 to <70 years (Table 6). Additionally, those in this age group were not more likely to report their economic situation had worsened since summer 2019 than those aged ≥50 to <70 years. However, they were more likely to be concerned about their future economic circumstances than those aged ≥50 to <70 years (Table 5). In this age group, 26.6% reported a decline in mental health in the last year, compared to just over 1 in 10 among those aged ≥70 (Table 3).

Whilst people aged ≥50 to <70 years were not significantly more likely to report a moderate or severe impact of the pandemic compared to those aged 70 years and over (Table 6), they were more likely to be concerned about their about both their future professional and economic circumstances than adults those aged ≥70 years (Table 5). In this age group, the greatest proportion (27.0%) reported a decline in mental health, similar to those aged ≥35 to <50 years (Table 3). They were 1.85 times as likely to report a decline in mental health compared to the youngest age group (18 to <35, 95% CI: 1.16, 2.96) and 3.41 times as likely to report a decline compared to those aged ≥70 (95% CI: 1.37, 8.51).

People aged 70 and older were least likely to be concerned about their future economic circumstances, and least likely to report a decline in mental health. As expected, the oldest age group (≥70 years) were significantly more likely to report a decline in physical health (OR 2.44 (95% CI: 1.08, 5.54) Table 4).

Age group was not associated with changes in smoking and physical activity.

#### 3.3.2 Sex

Women were nearly 40% more likely to report a moderate or severe overall effect of the pandemic than men (Table 6). Whilst no differences were found in a decline in SRPH by sex, women were more likely to report a decline in mental health (OR 1.84 (95% CI: 1.29, 2.64), Table 4).

Sex was not significantly associated with any changes in smoking, alcohol consumption or physical activity in the last year (Web Appendix 5). There were no significant associations between sex and changes in professional or economic circumstances, nor in concern for these in the future.

#### 3.3.3 Working in tourism

People working in the tourism sector were nearly 50% more likely to report a moderate or severe impact of the pandemic than others (OR 1.47 (95% CI: 1.00, 2.15) Model 2, Table 6). This effect was no longer significant when controlling for changes and concern for economic circumstances (Model 3 and 4, Table 6). A person working in tourism was seven times more likely to report worsening in their professional situation and nearly three times as likely to report worsening in their financial situation than people working in other sectors (Table 5). Tourism workers were more likely be concerned for both their future professional activity and finances—even when controlling for any decline in economic circumstances since summer 2019 (Web Appendix 7).

People working in tourism were twice as likely to report a decline in SRMH [OR 2.03 (95% CI: 1.26, 3.27)] although the effect was attenuated when controlling for worsening financial and professional status [OR 1.68 (95% CI: 1.01, 2.79)] and became non-significant when additionally controlling for professional and financial concerns [OR 1.26 (95% CI: 0.74, 2.14)], indicating a clear nexus between mental health and economic effects.

No differences were found between those working in tourism and others in changes in SRPH, smoking, alcohol consumption or physical activity.

## 4 Discussion

The present study examines the association between prior infection with SARS-CoV-2 and wide-ranging impacts of the COVID-19 pandemic, including changes in self-rated physical and mental health, behaviours and economic status. It also analysed disparities in outcomes by sociodemographic characteristics.

### 4.1 Main findings

We found that seropositivity was associated with indicators of worsened physical health (although absolute levels were low) and certain symptoms in the subsequent months, but not changes in behaviours.

Combining the ≥50 to <70 and ≥70 age groups, 11.0% reported worsened SRPH, similar to the Austrian SHARE study (10.9% of those over 50), which compared pre-SARS-CoV-2 data to June 2020. Though using a different time frame and a three-point response scale (28), both studies align. Despite the low absolute prevalence of poor physical health in our study, it was linked to seropositivity, suggesting a limited but lasting impact of SARS-CoV-2 on self-reported health.

Behavioural changes were even more limited than those in self-reported physical health and were unrelated to seropositivity. Few changes were noted; smoking increased more than it decreased, while alcohol consumption declined and physical activity rose. The drop in alcohol use aligns with NPI-related bar closures, but increased physical activity contrasts with most studies showing declines (29). his may reflect altered work patterns and unique access to the Alps in Ischgl, allowing for socially distanced exercise. Differences in pre-pandemic activity norms, like Iceland’s reliance on team sports, and study design variations may also explain these trends (30).

Despite stable behaviours and modest health declines, 1 in 2 participants reported a moderate or severe pandemic impact, mainly on mental health and finances—both unrelated to seropositivity. This underscores the strong, lasting indirect effects of the pandemic.

By November 2020, 8 months into the pandemic, 15.7% of participants reported poor physical or mental health. Over 20% saw a mental health decline since November 2019, with few improving, while 10% reported worse physical health. In August 2019, the OECD found 7.8% of Austrians aged 15+ had poor SRH. Though that study did not separate physical and mental health, our findings suggest mental health decline was a major pandemic impact (31).

A March–April 2020 study in Austria found 43% of those over 16 reported moderate or severe psychological impact, with higher rates in women (6). Our study, conducted in November 2020, found lower rates of poor SRMH (13.4%) and SRMH decline (23.3%). Differences may stem from study design, bias, and timing. Early 2020 saw strict NPIs, while by November, the population had adapted, possibly easing mental health effects despite Ischgl’s second wave. The NAKO study linked lower psychological distress to lower incidence rates, and Ischgl’s relatively few new infections may have mitigated mental health declines (23).

The most prevalent impact of the pandemic in our study population was economic; 50% of people reported a worsening of their professional and/or financial situation. This did not differ by seropositivity. However, certain segments of the population were disproportionately affected by particular impacts of the pandemic.

Tourism workers faced the greatest professional and financial impact due to NPIs, reflecting the sector’s local importance. Early in the pandemic, the economic effects of NPIs were unclear. By October 2020, a Tyrolean government report estimated the early ski season closure cut Ischgl’s seasonal revenue by 20–30% (17), despite mitigation efforts. Our study supports the well-known link between economic strain and mental health. Tourism workers had higher SRMH decline rates from November 2019–2020, explained by financial and job pressures. Similar findings were reported in Germany’s NAKO study (32).

Other population groups were also disproportionately affected by the pandemic—with differential effects by age and sex, in line with other studies in other European populations, and in Austria (6, 33). Overall, those aged 18 to <35 years who were most likely to report a moderate or severe overall impact effect of the pandemic, despite older age groups being at greatest risk for serious illness and mortality caused by COVID-19. For example, at Ischgl-1, only 2% of all participants found to be seropositive had been hospitalised because of COVID-19, yet 14.8% of seropositive participants aged over 70 were hospitalised. In November 2020, the oldest age group was more likely to report declines in SRPH since November 2019 than other age groups, but these declines did not appear to be directly exacerbated by SARS-CoV-2 infection. In a stratified analysis, no relationship was found between seropositivity and a decline in SRPH among this age group. However, those over 70 years of age were far less likely to be concerned about their economic future than the other age groups, and less likely to report any declines in mental health. Declines in mental health and current poor MH were most acute in the middle age groups (≥35 to <50 and ≥ to <70 years) which is in line with the SHARE study when comparing to older age groups (34).

Women were more likely to report a moderate or severe effect of the pandemic than men. In line with the Traunmüller study in Austria, we also found women reported more worsening of their mental health (6). Of the 13% of participants reporting poor SRMH, the majority were women, in line with pre-pandemic studies which have consistently shown women tend to report higher rates of psychological distress than men, reasons for which remain unclear conclusive but have been linked to differences in exposure to risks for developing, vulnerabilities to, experiences of, and responses to mental health between genders (6, 35). Women in our study were also more likely to report declines in their mental health than men. Other studies have linked the disproportionate effect of the pandemic on women’s mental health to be associated with changes in social networks, social activities and contact and social isolation, alongside changes in care-giving responsibilities including a greater responsibility for homeschooling during lockdowns, despite potential other benefits from working from home (10, 36). Further research would be needed to understand these factors in the Austrian context, and it is important to highlight our study lacks data on wider social networks that are clearly integral to both the wider impacts of NPIs and understanding how NPIs impact people. Indeed, the fact that our study population experienced the same NPIs and there were no gender differences in laboratory-confirmed seropositivity suggests it is not NPIs or SARS-CoV-2 infection, per se, that has led to this disparity.

### 4.2 Strengths and limitations

The strength of this study is that it is based on a population with laboratory-confirmed SARS-CoV-2 status, all infected in the same outbreak and experiencing the same waves of the pandemic and NPIs. Nonetheless, it has some limitations. It relies on self-reported data, which introduces the possibility of recall bias concerning their health, behaviours, or economic status. Also, while the situation in Ischgl provides an unusual opportunity to undertake such a study, as a community heavily reliant on tourism the generalisability of the findings is limited. Another limitation stems from the timing of data collection. The follow-up survey occurred in November 2020, during the second wave of the COVID-19 pandemic, and will not capture the long-term effects of the virus or the non-pharmaceutical interventions used to control its spread. Also, while we examined some behaviours directly related to health, there may be others, such as gambling, that could have been impacted by the pandemic. Our health assessments relied on single-item measures for physical and mental health, which, while widely used, may not fully capture the complexity of health changes experienced by participants. Additionally, variations in the intensity and timing of infection waves and NPIs within Austria could introduce variability not fully addressed in the analysis. Finally, we lacked data on social networks, which may be relevant given the impact of social connectivity on mental health during periods of restrictive measures.

### 4.3 Recommendations

This study reinforces recommendations that have been made by others in the light of experience during the pandemic. Mental health emerged as a critical concern, particularly for women and middle-aged adults, many of whom reported declines in well-being. Pandemic preparedness should include the ability to scale up online counselling and other forms of remote support, some of which could be delivered by volunteers with basic training and appropriate safeguards. Economic strain, especially for workers in tourism-reliant regions like Ischgl, highlights the need for financial aid and job retraining programmes that can be deployed during and after a pandemic. Looking ahead, especially in the light of climate change and its impact on snow cover, diversifying local economies in ski resorts will offer longer-term stability. For younger adults who expressed concerns about job security, initiatives like internships, apprenticeships, and guaranteed job opportunities can help stabilise their professional trajectories. There is a clear need to monitor individuals with persistent physical symptoms.

Preparedness for future pandemics must include tailoring non-pharmaceutical interventions to specific community contexts, aiming to minimise mental health and economic impacts. Collecting appropriate data on social, psychological, and economic outcomes during health crises is vital for holistic response plans. Gender-sensitive policies should also be prioritized to address the disproportionate burdens on women, such as expanded childcare support, flexible work arrangements, and mental health initiatives tailored to their needs.

## 5 Conclusion

Whilst the effects of the pandemic reported by participants in Ischgl were wide-ranging, the population of Ischgl was relatively resilient to the impact of the pandemic in terms of physical health and behaviours. However, half of the population considered the overall effect of the pandemic to be moderate or severe by November 2020. Considerable strain was apparent among the economically active population. Women and middle-aged participants were disproportionality affected in terms of their mental health. Furthermore, a clear nexus exists between economic pressures and declines in mental health, as reflected in the concerns of those working in the tourism sector, which dominates the economy of Ischgl. In future pandemics particular attention should be given to communities built on tourism, but the experience of Ischgl highlights that such populations, supported by appropriate government policies, can be resilient and recover well.

There are idiosyncrasies in the experience of Ischgl during the COVID-19 pandemic—which highlight the importance of context in pandemic preparedness. Equally, many impacts in Ischgl were seen in other European studies. Whilst many countries worldwide were able to use modelling to inform measures to prevent deaths during the pandemic, it is widely accepted that, in most countries, evidence on the social and economic impacts of mitigation policies were less reliable and available (37). The provision of timely and robust data detailing the wider aspects of the impact during these crucial phases, particularly social and economic aspects, should be a priority in future pandemic preparedness, aiming to reduce the length and depth of the indirect effects of a pandemic.

## Data Availability

The raw data supporting the conclusions of this article will be made available by the authors, without undue reservation.
