# Supplementary material for: Changes in self-reported physical and mental health, behaviour and economic status among adults by known seropositivity and sociodemographic factors before and after the COVID-19 pandemic outbreak in Ischgl, Austria
Source: Front Public Health. 2025 Apr 2;13:1488108. doi: 10.3389/fpubh.2025.1488108 (PMC12002084; doi:10.3389/fpubh.2025.1488108)
Supplement: Supplementary file 1 [file Table_1.docx]

**Web Appendix 1: Descriptive Statistics for Self-Reported Physical and Mental Health at Ischgl-2**

|  | Poor Self Rated physical Health at Ischgl-2* | | p-value | Poor Self-rated Mental Health* at Ischgl-2 | | p-value |
| --- | --- | --- | --- | --- | --- | --- |
|  | Poor | Other |  | Poor | Other |  |
| **Total,** n (%) | | | | | | |
|  | 37 (4.6) | 763 (95.4) |  | 107 (13.4) | 693 (86.6) |  |
| **Seropositivity at Ischgl-1,** n (%) | | | | | | |
| Seropositive | 24 (6.0) | 379 (94.0) | 0.071 | 53 (13.2) | 350 (86.9) | 0.851 |
| Seronegative | 13 (3.3) | 384 (96.7) |  | 54 (13.6) | 343 (86.4) |  |
| **Sex,** n (%) | | | | | | |
| Female | 23 (5.2) | 416 (94.8) | 0.362 | 76 (17.3) | 363 (82.7) | <0.001 |
| Male | 14 (3.9) | 347 (96.1) |  | 31 (8.6) | 330 (91.4) |  |
| **Age Groups (years),** n (%) | | | | | | |
| 18 to <35 | 5 (2.2) | 223 (97.8) | <0.001^¥^ | 18 (7.9) | 210 (92.1) | 0.003^¥^ |
| ≥35 to <50 | 3 (1.3) | 226 (98.7) |  | 27 (11.8) | 202 (88.2) |  |
| ≥50 to <70 | 17 (6.0) | 268 (94.0) |  | 54 (19.0) | 231 (81.1) |  |
| ≥70 | 12 (20.7) | 46 (79.3) |  | 8 (13.8) | 50 (86.2) |  |
| **Age group (>/< Median),** n (%) | | | | | | |
| ≥45 | 29 (7.0) | 386 (93.0) | <0.001 | 70 (16.9) | 345 (83.1) | 0.003 |
| < 45 | 8 (2.1) | 377 (97.9) |  | 37 (9.61) | 348 (90.4) |  |
| **BMI (groups)** †, n (%) | | | | | | |
| <25.0 | 20 (4.3) | 445 (95.7) | 0.378 | 69 (14.8) | 396 (85.2) | 0.164 |
| ≥25.0 to <30.0 | 10 (4.0) | 241 (96.0) |  | 25 (10.0) | 226 (90.0) |  |
| ≥30.0 | 6 (7.6) | 73 (92.4) |  | 12 (15.2) | 67 (84.8) |  |
| **Morbidity†**† **,** n (%) | | | | | | |
| No (%) | 15 (2.5) | 579 (97.5) | <0.001 | 72 (12.1) | 522 (87.9) | 0.073 |
| Yes (%) | 22 (10.7) | 183 (89.3) |  | 35 (17.1) | 170 (82.9) |  |
| **Self-rated Mental Health*,** n (%) | | | | | | |
| Good | 19 (2.74) | 674 (97.3) | <0.001 | 89 (11.7) | 674 (88.3) | <0.001 |
| Poor | 18 (16.8) | 89 (83.2) |  | 18 (48.7) | 19 (51.4) |  |
| **Decline in Physical Health *,** n (%) | | | | | | |
| No | 13 (1.8) | 718 (98.2) | <0.001 | 87 (11.9) | 644 (88.1) | <0.001 |
| Yes | 24 (34.8) | 45 (65.2) |  | 20 (29.0) | 49 (71.0) |  |
| **Decline in Mental Health,** n (%) | | | | | | |
| No | 19 (3.1) | 595 (96.9) | <0.001 | 12 (2.0) | 602 (98.1) | <0.001 |
| Yes | 18 (9.7) | 168 (90.3) |  | 95 (51.1) | 91 (48.9) |  |

^¥^n=800, †n=795, **†**†n=799 ^fisher’s exact p-value

**Web Appendix 2: Adjusted Odds Ratios for reporting poor SRPH and poor SRMH at Ischgl-2**

|  | OR reporting poor SRPH at Ischgl-2 (95% CI) | OR reporting poor SRMH at Ischgl-2 (95% CI) |
| --- | --- | --- |
| **Seropositivity** *(ref: Seronegative)*  Seropositive | 2.38* (1.14,5.01) | 1.00 (0.65,1.52) |
| **Sex** *(ref: Male)*  Female | 1.38 (0.65,2.92) | 2.14** (1.34,3.42) |
| **Age** *(ref: ≥50 to <70 years)* |  |  |
| 18 to <35 years  ≥35 to <50 years | 0.42 (0.14,1.27)  0.28 (0.08,1.01) | 0.36** (0.19,0.66)  0.59 (0.34,1.02) |
| ≥70 years | 2.82* (1.16,6.88) | 0.65 (0.28,1.51) |
| **BMI kg/m^2^***(ref: 25.0-30.0)* |  |  |
| <25.0 | 1.48 (0.64,3.43) | 1.61 (0.96,2.70) |
| >30.0 | 1.42 (0.47,4,24) | 1.41 (0.66,3.01) |
| **Morbidity** *(ref: None)* |  |  |
| Yes | 2.42* (1.08,5.40) | 1.46 (0.88,2.42) |
| **Education** (*ref: Compulsory)*  Other  High School  University | 2.02 (0.49,8.29)  0.73 (0.32,1.68)  1.23 (0.35,4.31) | 1.18 (0.37,3.82)  1.12 (0.67,1.87)  0.74 (0.30,1.83) |
| N | 794 | 794 |

* *p* < 0.05, ** *p* < 0.01, *** *p* < 0.001

**Web Appendix 3 Descriptive Statistics for Current Health Behaviours**

|  | **Currently Smokes^** | | **p-value** | **Consumes Alcohol** | | **p-value** | **Currently Exercises** | | **p-value** |
| --- | --- | --- | --- | --- | --- | --- | --- | --- | --- |
|  | **No** | **Yes** |  | **No** | **Yes** |  | **No** | **Yes** |  |
| **Total (n)** | 539 (67.7) | 257 (32.3) |  | 136 (17.0) | 664 (83.0) |  | 109 (13.6) | 691 (88.3) |  |
| **Sex n(%)** | | | | | | | | | |
| Female | 299 (68.7) | 136 (31.3) | 0.498 | 102 (23.2) | 337 (76.8) | <0.001 | 74 (16.8) | 366 (83.2) | 0.003 |
| Male | 240 (66.5) | 121 (33.5) |  | 34 (9.4) | 327 (90.6) |  | 35 (9.7) | 326 (90.3) |  |
| **Age group (years)** | | | | | | | | | |
| ≥18 to <35 | 136 (59.7) | 92 (40.4) | 0.002 | 16 (7.0) | 212 (93.0) | <0.001 | 27 (11.8) | 201 (88.2) | 0.101 |
| ≥35 to <50 | 158 (69.9) | 68 (30.1) |  | 30 (13.1) | 199 (86.9) |  | 38 (16.6) | 191 (83.4) |  |
| ≥50 to < 70 | 197 (69.1) | 88 (30.9) |  | 63 (22.1) | 222 (77.9) |  | 21 (11.2) | 254 (88.8) |  |
| ≥70+ | 48 (84.2) | 9 (15.8) |  | 27 (46.6) | 31 (53.5) |  | 12 (20.7) | 46 (79.3) |  |
| **Morbidity ^^** | | | | | | | | | |
| No | 386 (65.4) | 204 (34.6) | 0.021 | 83 (14.0) | 511 (86.0) | <0.001 | 77 (13.0) | 517 (87.0) | 0.341 |
| Yes | 152 (74.2) | 53 (25.9) |  | 52 (25.4) | 153 (74.6) |  | 32 (15.6) | 173 (84.4) |  |
| **BMI kg/m^2^  ^^^** | | | | | | | | | |
| <25 | 295 (63.7) | 168 (36.3) | 0.011 | 68 (14.6) | 397 (85.4) | 0.132 | 54 (11.6) | 411 (88.4) | 0.049 |
| ≥25 to <30 | 179 (71.3) | 72 (28.7) |  | 51 (20.3) | 200 (79.7) |  | 37 (14.7) | 215 (85.3) |  |
| ≥30 | 62 (78.5) | 17 (21.5) |  | 15 (19.0) | 64 (81.0) |  | 17 (21.5) | 62 (78.5) |  |
| **Education** | | | | | | | | | |
| Other | 20 (76.9) | 6 (23.1) | 0.019 | 7 (26.9) | 19 (73.1) | <0.001 | 0 (0.0) | 26 (100.0) | 0.005^¥^ |
| Compulsory | 139 (74.7) | 47 (25.3) |  | 51 (27.3) | 136 (72.7) |  | 38 (20.3) | 149 (79.7) |  |
| High School | 320 (63.8) | 182 (36.3) |  | 71 (14.1) | 433 (85.9) |  | 61 (12.1) | 443 (87.9) |  |
| University | 69 (73.2) | 22 (26.8) |  | 7 (8.4) | 76 (91.6) |  | 10 (12.1) | 73 (88.0) |  |
| **Seropositivity** | | | |  |  |  |  |  |  |
| Positive | 292 (72.8) | 109 (27.2) | 0.002 | 72 (17.9) | 331 (82.1) | 0.511 | 47 (11.7) | 356 (88.3) | 0.106 |
| Negative | 247 (62.5) | 148 (37.5) |  | 64 (16.1) | 333 (83.9) |  | 62 (15.6) | 336 (84.4) |  |
| **Self reported Physical Health †** | | | | | | | | | |
| Poor | 30 (81.1) | 7 (18.9) | 0.075 | 11 (29.7) | 26 (70.3) | 0.035 | 7 (18.9) | 30 (81.1) | 0.336 |
| Other | 509 (97.1) | 250 (32.9) |  | 125 (16.4) | 638 (83.6) |  | 102 (13.4) | 661 (86.6) |  |
| **Decline in Self reported Physical Health †** | | | | | | | | | |
| Yes | 57 (82.6) | 12 (17.4) | 0.006 | 11 (15.9) | 58 (84.1) | 0.807 | 10 (14.5) | 59 (85.5) | 0.826 |
| No | 482 (66.3) | 245 (33.7) |  | 125 (17.1) | 606 (82.9) |  | 99 (13.5) | 632 (86.5) |  |
| **Self reported Mental Health †** | | | | | | | | | |
| Poor | 67 (63.2) | 39 (36.8) | 0.287 | 26 (24.3) | 81 (75.7) | 0.031 | 17 (15.9) | 90 (84.1) | 0.463 |
| Other | 472 (68.4) | 218 (31.6) |  | 110 (15.9) | 583 (84.1) |  | 92 (13.3) | 601 (86.7) |  |
| **Decline in Self reported Mental Health †** | | | | | | | | | |
| Yes | 118 (63.8) | 67 (36.2) | 0.192 | 35 (18.8) | 151 (81.2) | 0.451 | 26 (14.0) | 160 (86.0) | 0.873 |
| No | 421 (68.9) | 190 (31.1) |  | 101 (16.5) | 513 (83.6) |  | 83 (13.5) | 531 (86.5) |  |

^n=796, ^^n=795, ^^^n=793, †n=800, ^¥^fisher’s exact

**Web Appendix 4: Logistic Regression Model Results for Current Health Behaviours (Odds Ratios, ORs)**

|  | **Current Smoker**  **(ref: never /former)**  **OR** | **Current Smoker**  **(ref: never /former)**  **OR** | **Consumes Alcohol**  **(ref: never/former)**  **OR** | **Consumes Alcohol**  **(ref: never/former)**  **OR** | **Exercises**  **(ref: does not exercise)**  **OR** | **Exercises**  **(ref: does not exercise)**  **OR** |
| --- | --- | --- | --- | --- | --- | --- |
| **Seropositivity at Ischgl-1 (ref: *seronegative*)** | | | | | | |
| Seropositive | 0.62** (0.45,0.84) | 0.62** (0.45,0.85) | 0.77 (0.51,1.15) | 0.78 (0.52,1.16) | 1.42 (0.93,2.17) | 1.43 (0.93,2.19) |
| **Gender (ref: male)** |  |  |  |  |  |  |
| Female | 0.81 (0.59,1.12) | 0.81 (0.58,1.11) | 0.31*** (0.20,0.49) | 0.30***(0.19,0.47) | 0.50 (0.32,0.79) | 0.50** (0.32,0.79) |
| **Age group (ref ≥50 to <70 year)** |  |  |  |  |  |  |
| ≥18 to <35 years | 1.33 (0.88,2.00) | 1.34 (0.89, 2.03) | 3.05*** (1.63,5.71) | 2.98** (1.59,5.58) | 0.61 (0.33,1.13) | 0.60 (0.32,1.12) |
| ≥ 35 to <50 years | 0.86 (0.57,1.30) | 0.87 (0.58,1.32) | 1.62 (0.96,2.73) | 1.58 (0.93,2.67) | 0.48* (0.27,0.85) | 0.47** (0.27,0.83) |
| ≥70 years | 0.57 (0.26,1.26) | 0.57 (0.26, 1.27) | 0.36* (0.18,0.70) | 0.37** (0.19,0.73) | 0.51 (0.23,1.15) | 0.54 (0.24,1.22) |
| **Morbidity (ref: no)** |  |  |  |  |  |  |
| Yes | 0.89 (0.59,1.34) | 0.89 (0.59,1.34) | 0.79 (0.48,1.27) | 0.79 (0.48,1.28) | 0.83 (0.48,1.44) | 0.84 (0.49,1.45) |
| **BMI g/km2 (ref <25.0)** |  |  |  |  |  |  |
| ≥25.0 to <30.0 | 1.33 *0.93,1.91) | 1.32 (0.92,1.90) | 1.39 (0.88,2.19) | 1.40 (0.89, 2.21) | 1.43 (0.88,2.32) | 1.44 (0.89,2.35) |
| ≥30.0 | 0.75 (0.40,1.40) | 0.74 (0.40,1.38) | 1.18 (0.59,2.36) | 1.21 (0.60,2.42) | 0.63 (0.32,1.23) | 0.64 (0.32,1.25) |
| **Education** (*ref: Compulsory)* |  |  |  |  |  |  |
| Other | 0.74 (0.27,2.00) | 0.75 (0.28,2.03) | 0.77 (0.28,2.13) | 0.78 (0.28,2.16) | - | - |
| High School | 1.36 (0.94,2.14) | 1.43 (0.95,2.16) | 1.37 (0.86,2.18) | 1.35 (0.85,2.15) | 1.88* (1.14,3.11) | 1.86* (1.12,3.08) |
| University | 0.82 (0.44,1.54) | 0.84 (0.45,1.57) | 1.89 (0.78,4.60) | 1.88 (0.77,4.60) | 1.82 (0.32,1.23) | 1.80 (0.80,4.02) |
| **Decline in PH (ref: no)** |  |  |  |  |  |  |
| Yes | 0.48* (0.25,0.94) | 0.49 (0.24,1.01) | 1.79 (0.28,2.13) | 2.00 (0.85,4.73) | 1.06 (0.50,2.25) | 1.23 (0.53,2.86) |
| **Decline in MH (ref no)** |  |  |  |  |  |  |
| Yes | 1.36 (0.94,1.96) | 1.21 (0.77,1.92) | 0.80 (0.83,3.83) | 0.93 (0.51,2.15) | 0.92 (0.56,1.53) | 1.00 (0.52,1.91) |
| **Self-Rated Physical Health**  **(ref: Good)** |  |  |  |  |  |  |
| Average/Poor | - | 0.88 (0.33,2.36) | - | 0.77 (0.28,2.11) | - | 0.63 (0.21,1.86) |
| **Self-Rated Mental Health**  **(ref: Good)** |  |  |  |  |  |  |
| Average/Poor | - | 1.28 (0.72,2.29) | - | 0.77 (0.38,1.56) | - | 0.89 (0.41,1.94) |
| N | 792 | 792 |  | 794 | 768 | 768 |

* *p* < 0.05, ** *p* < 0.01, *** *p* < 0.001

**Web Appendix 5: Multinomial Regression Model Results for Change in Health Behaviours since Ischgl-1 (Relative Risk Ratios, RRs)**

|  | **Change in Smoking**  **(ref: no change)**  **RR** | | **Change in Alcohol Consumption**  **(ref: no change)**  **RR** | | **Change in Exercise**  **(ref: no change)**  **RR** | |
| --- | --- | --- | --- | --- | --- | --- |
|  | **Less** | **More** | **Less** | **More** | **Less** | **More** |
| **Sex (ref: male)** | | | | | | |
| Female | 0.98 (0.31,3.06) | 0.66 (0.31,1.41) | 0.68 (0.38,1.21) | 2.08 (0.93,4.64) | 0.96 (0.55,1.68) | 0.83 (0.54,1.26) |
| **Age group**  **(ref: ≥ 50 to <70 years)** | | | | | | |
| ≥18 to <35 years | 1.33 (0.39,4.52) | 1.89 (0.72,4.95) | 3.28* (1.49,7.24) | 2.08 (0.93,4.64) | 0.92 (0.43,1.97) | 1.32 (0.78,2.22) |
| ≥ 35 to <50 years | - | 1.35 (0.49,3.68) | 0.94 (0.37,2.39) | 0.84 (0.33,2.14) | 1.27 (0.64,2.53) | 0.86 (0.50,1.49) |
| ≥70 years | 5.40 (0.63,45.60) | 1.80 (0.17,18.72) | 1.81 (0.35,9.42) | 3.80 (0.84,17.07) | 1.37 (0.49,3.87) | 0.48 (0.14,1.70) |
| **Seropositivity at Ischgl-1**  **(ref: seronegative)** | | | | | | |
| Seropositive | 1.45 (0.48,4.42) | 1.41 (0.67,2.96) | 0.86 (0.49,1.52) | 1.74 (0.83,3.64) | 0.62 (0.36,1.07) | 0.88 (0.58,1.32) |
| **BMI (ref: <25.0 kg/m2)** |  |  |  |  |  |  |
| BMI ≥25.0 - <30.0 | 3.89 (0.77,19.66) | 1.94 (0.76,4.92) | 1.46 (0.72,2.95) | 1.04 (0.45, 2.41) | 0.61 (0.33,1.11) | 1.02 (0.63,1.66) |
| BMI ≥30.0 | - | 1.43 (0.26,8.01) | 0.79 (0.21,2.95) | 0.25 (0.03,2.12) | 0.42 (0.133,1.35) | 1.27 (0.60,2.71) |
| **Morbidity (ref: no)** |  |  |  |  |  |  |
| Yes | 0.94 (0.22, 3.93) | 0.86 (0.30,2.48) | 0.70 (0.29,1.68) | 0.65 (0.25,1.74) | 2.62 (0.74,9.28) | 0.72 (0.41,1.26) |
| **Education** (*ref: Compulsory)* |  |  |  |  |  |  |
| Other | 2.83 (0.19,41.38) | 1.28 (0.11,14.36) | 1.60 (0.28,9.23) | 0.76 (0.08,6.88) | 2.62 (0.74,9.28) | 1.55 (0.50,4.81) |
| High School | 0.70 (0.16,3.07) | 0.69 (0.26,1.84) | 1.44 (0.57,3.66) | 0.63 (0.25,1.57) | 1.26 (0.61,2.62) | 1.18 (0.68,2.07) |
| University | 1.62 (0.25,10.52) | 0.52 (0.09,2.93) | 1.27 (0.39,4.19) | 2.21 (0.72,6.80) | 2.39 (0.91,6.28) | 1.40 (0.64,3.09) |
| **Decline in Physical Health**  **(ref: no)** |  |  |  |  |  |  |
| Decline | 1.83 (0.19,18.1) | 1.42 (0.28,7.30) | 1.43 (0.52,3.96) | 1.36 (0.49,3.78) | 6.44*** (3.29,12.62) | 0.63 (0.23,1.70) |
| **Decline in Mental Health**  **(ref: no)** |  |  |  |  |  |  |
| Decline | 0.67 (0.17,2.74) | 1.22 (0.53,2.83) | 0.77 (0.36,1.67) | 3.41** (1.60,7.23) | 1.78 (0.98,3.25) | 1.57 (0.97,2.52) |
| N | 257 | | 661 | | 686 | |

Note: BMI ≥30.0 excluded from Smoking due to small n; * *p* < 0.05, ** *p* < 0.01, *** *p* < 0.001

**Web Appendix 6: Logistic Regression Model Results for Specific Symptoms (Odds Ratios, OR)**

|  | **Nasal Symptoms**  **(ref: no)**  **OR** | **Throat**  **(ref: no)**  **OR** | **Loss tase and/or smell**  **(ref: no)**  **OR** | **Tight Chest**  **(ref: no)**  **OR** | **Fatigue**  **(ref: no)**  **OR** |
| --- | --- | --- | --- | --- | --- |
| **Seropositivity at Ischgl-1**  **(ref: seronegative)** | | | | | |
| Seropositive | 0.60** (0.42,0.84) | 0.56* (0.35,0.90) | 5.73*** (2.16,15.22) | 2.91* (1.20,7.06) | 2.07* (1.11,3.86) |
| **Gender (ref: male)** | | | | | |
| Female | 0.83 (0.58,1.17) | 1.25 (0.77,2.03) | 1.05 (0.49,2.25) | 22.38 (0.99,5.68) | 1.32 (0.72,2.44) |
| **Age group (ref ≥50 to <70 years)** | | | | | |
| ≥18 to <35 years | 2.80*** (1.80,4.36) | 2.63*** (1.41,4.92) | 0.81 (0.30,2.19) | 2.08 (0.68,6.41) | 3.20** (1.39,7.33) |
| ≥ 35 to <50 years | 1.29 (0.80,2.06) | 1.88 (0.97,3.63) | 1.02 (0.39,2.67) | 1.62 (0.53,4.99) | 1.70 (0.71,4.10) |
| ≥70 years | 1.29 (0.59,2.83) | 1.20 (0.37,3.82) | 1.92 (0.52,7.01) | 2.38 (0.64,8.90) | 1.96 (0.63,6.09) |
| **BMI (ref: <25.0 kg/m2)** | | | | | |
| BMI ≥25.0 - <30.0 | 0.76 (0.50,1.15) | 0.75 (0.42,1.34) | 0.30* (0.11,0.82) | 2.02 (0.82,4.97) | 1.36 (0.68,2.71) |
| BMI ≥30.0 | 1.86* (1.06,3.28) | 1.11 (0.49,2.54) | 0.30 (0.07,1.39) | 1.47 (0.38,5.68) | 1.47 (0.56,3.90) |
| **Morbidity (ref: no)** | | | | | |
| Yes | 0.77 (0.48,1.23) | 0.96 (0.50,1.83) | 1.79 (0.75,4.31) | 1.88 (0.73,4.85) | 1.81 (0.88,3.74) |
| N | 794 | 794 | 794 | 794 | 794 |

* *p* < 0.05, ** *p* < 0.01, *** *p* < 0.001

**Web Appendix 7: Logistic Regression Model Results for Concern about future professional situation and financial situation, controlling for worsened professional and financial circumstances, respectively (Odds Ratio, OR)**

|  | **OR for concern about future professional situation, ref: not concerned** | **OR for concern about future financial situation, ref: not concerned** |  |
| --- | --- | --- | --- |
|  |  |  |  |
| **Seropositivity at Ischgl 1** | | |  |
| Seropositive | 0.64** (0.46,0.89) | 1.00 (0.72, 1.38) |  |
| **Education (ref: compulsory)** | | |  |
| Other | 0.52 (0.19,1.49) | 0.41 (0.15,1.11) |  |
| High School | 1.07 (0.70,1.63) | 0.88 (0.57, 1.34) |  |
| University | 0.78 (0.41,1.48) | 0.50* (0.26,0.95) |  |
| **Tourism sector** | | |  |
| Yes | 3.998*** (2.54,6.26) | 2.48*** (1.64,3.74) |  |
| **Sex** | | |  |
| Female | 1.27 (0.90,1,79) | 1.37 (0.98,1.93)) |  |
| **Age Groups years (≥50 to <70 years)** | | |  |
| ≥18 to <35 years | 1.74* (1.12,2.71) | 1.19 (0.77,1.83) |  |
| ≥ 35 to <50 years | 2.30*** (1.50,3.54) | 1.89** (1.23,2.92) |  |
| ≥70 years | 0.15** (0.04,0.55) | 0.21** (0.08,0.53) |  |
| **Morbidity†**† | | |  |
| Yes | 1.00 (0.65,1.55) | 1.11 (0.72,1.70) |  |
| **Self-rated Physical Health** | | |  |
| Poor | 0.49 (0.18,1.35) | 0.85 (0.32,2.24) |  |
| **Self-rated Mental Health** | | |  |
| Poor | 1.35 (0.71,2.59) | 1.17 (0.60,2.28) |  |
| **Decline in Physical Health** | | |  |
| Yes | 1.08 (0.55,2.12) | 0.94 (0.47,1.87) |  |
| **Decline in Mental Health** | | |  |
| Yes | 2.52*** (1.52,4.18) | 2.28** (1.35,3.84) |  |
| **Professional Situation Worsened** | | |  |
| Yes | 3.44*** (2.32,5.11) |  |  |
| **Financial Situation Worsened** | | |  |
| Yes |  | 3.11*** (2.21,4.37) |  |
| N | 788 | 759 |  |

* *p* < 0.05, ** *p* < 0.01, *** *p* < 0.001

**Web Appendix 8: Descriptive Statistics for Overall Effect of the Pandemic**

| (n, (%))/Median (IQR) | No//Little Effect | Moderate/Severe Affect | p-value (chi2/Mann-Whitney U-test) |
| --- | --- | --- | --- |
| **Total** | | | |
| (n, (%))/ (IQR) | 353 (44.4) | 443 (55.6) |  |
| **Seropositivity at Ischgl-1** | | | |
| Seropositive | 163 (40.6) | 239 (59.5) | 0.027 |
| Seronegative | 191 (48.4) | 204 (51.7) |  |
| **Sex** | | | |
| Female | 178 (40.8) | 259 (59.2) | 0.025 |
| Male | 176 (48.8) | 185 (51.3) |  |
| **Education** |  |  |  |
| Other | 11 (42.3) | 15 (57.7) | 0.013 |
| Compulsory | 101 (54.9) | 83 (45.1) |  |
| High School | 209 (41.4) | 295 (58.5) |  |
| Higher Education | 33 (39.8) | 50 (60.2) |  |
| **Age Groups years** | | | |
| ≥18 to <35 | 84 (36.8) | 144 (63.2) | 0.003 |
| ≥35 to <50 | 94 (41.2) | 134 (58.8) |  |
| ≥50 to < 70 | 144 (50.5) | 141 (49.5) |  |
| ≥70+ | 32 (57.1) | 24 (42.9) |  |
| **Morbidity†**† | | | |
| No (%) | 260 (43.8) | 333 (56.2) | 0.543 |
| Yes (%) | 94 (46.3) | 109 (53.7) |  |
| **Self-rated Physical Health** | | | |
| Good | 341 (44.9) | 419 (55.1) | 0.245 |
| Poor | 13 (35.1) | 24 (64.9) |  |
| **Self-Rated Mental Health** | | | |
| No | 328 (47.5) | 362 (52.5) | <0.001 |
| Yes | 26 (24.3) | 81 (75.7) |  |
